# Burnout and Job Satisfaction Among Audiologists in Türkiye: A Multidimensional Assessment

**DOI:** 10.3390/healthcare14101363

**Published:** 2026-05-15

**Authors:** Uğur Belet, Tijen Zeybek

**Affiliations:** 1 Faculty of Health Science, Department of Audiology, Near East University, Nicosia 99138, Cyprus; 2 Faculty of Economics and Administrative Sciences, Department of Business Administration, Near East University, Nicosia 99138, Cyprus; tijen.zeybek@neu.edu.tr

**Keywords:** burnout syndrome, job satisfaction, audiologist

## Abstract

Objectives: This study aims to evaluate burnout and job satisfaction levels among audiologists in Türkiye and the factors that contribute to them. Methods: The study included 152 female (59.80%) and 102 male (40.20%) audiologists aged 20–30 (*n* = 133, 52.40%), 31–40 (*n* = 82, 32.30%), and 41 and over (*n* = 39, 15.40%). Participants were required to have at least 1 year of experience and be an active audiologist. Analysis was performed using Structural Equation Modeling (SEM) and correlation statistics to assess the predictive relationship between burnout sub-dimensions and job satisfaction components. Results: The results showed that burnout and job satisfaction among audiologists differed according to demographic data. In Türkiye, audiologists reported high burnout and low job satisfaction. A significant relationship was found between burnout and job satisfaction. The exhaustion subscale of the Maslach Burnout Scale statistically significantly and negatively predicted the Job Satisfaction Scale scores (β = −0.35; *p* < 0.05). Furthermore, it was determined that audiologists working in implant centers experienced less burnout, while those working in rehabilitation centers had lower job satisfaction. Conclusions: In conclusion, addressing burnout levels among audiologists is expected to not only improve individual well-being but also contribute to the overall enhancement of hearing health services in Türkiye.

## 1. Introduction

Burnout syndrome (BS) is a multidimensional psychological condition characterized by emotional exhaustion, depersonalization, and a diminished sense of professional competence [1]. Within the framework of the Maslach Burnout Inventory—General Form (MBI-GF), exhaustion refers to the depletion of mental energy, while depersonalization manifests as a negative, detached attitude toward work [2]. Furthermore, the third sub-dimension, competence, reflects an individual’s self-efficacy in their professional role. Employees experiencing emotional burnout often face difficulty connecting with their patients, leading them to withdraw emotionally and decrease productivity [3].

Conversely, job satisfaction (JS) represents the cognitive and affective evaluation of one’s work environment and professional experiences. Defined as a positive emotional state, JS is an integration of attitudes and cognitions stemming from various aspects of work, including career trajectory, administrative management, and physical working conditions [4]. It is a reflection of the level at which employees like or dislike their tasks, and it serves as a critical driver of employee motivation, performance, and the quality of interpersonal relationships [5].

The audiology profession occupies a unique position in this psychological landscape. As a specialized healthcare field, audiology requires a sophisticated combination of high-level technical expertise, managing complex digital equipment such as cochlear implants and hearing aids, and intense emotional labor [6]. Audiologists experiencing burnout often face difficulty connecting with their patients, leading them to withdraw emotionally a self-protective mechanism that can inadvertently result in negative attitudes, professional incompetence, and decreased productivity. Consequently, this state of professional inefficiency often manifests as guilt, lack of motivation, and persistent unhappiness in the workplace [7]. JS is essentially the psychological counterpart to the depletion observed in burnout, serving as a critical driver of motivation, performance, and the quality of interpersonal relationships. However, for the modern audiologist, JS is not merely a personal preference but a reflection of systemic support; it is deeply contingent upon the institutional environment, resource availability, and the clarity of professional roles within the clinic or hospital [8].

While the existing Turkish literature has addressed the professional challenges of audiologists by examining burnout and job satisfaction as isolated variables, often focusing narrowly on occupational stress levels or institution-specific differences, these studies remain largely descriptive and fragmented. This research bridges this significant theoretical gap by integrating these two fundamental factors into a comprehensive structural model that examines the predictive relationships between the sub-dimensions of burnout (exhaustion, depersonalization, and competence) and the key components of job satisfaction (career, management, and working conditions). By moving beyond simple correlations, this study contributes to the field by illustrating that a decline in job satisfaction is closely associated with specific dimensions of occupational burnout. Our findings suggest a significant link, indicating that job satisfaction levels may reflect the cumulative impact of burnout components within the audiological workforce. Ultimately, by shifting the discourse from descriptive individual analysis to a predictive systemic framework, this study offers the critical evidence-based insights necessary for institutional reform, addressing a void in the current audiology literature.

## 2. Methodology

### 2.1. Research Model

This study follows a cross-sectional analytical design aimed at exploring the predictive relationships between occupational burnout and job satisfaction. The research was approved by the Near East University Scientific Research Ethics Committee (YDU/2023/117-1775).

### 2.2. Working Group

The target population consisted of audiologists in Türkiye. A non-probability convenience and snowball sampling approach was employed. Participants were selected based on the following eligibility criteria: at least one year of professional experience and being currently active as an audiologist. The sample comprised 254 audiologists (152 women, 59.80%; 102 men, 40.20%). The participants’ demographic distribution is summarized in (Table 1). To ensure a comprehensive evaluation of the heterogeneous working environments in Türkiye, the study included audiologists from hospitals, hearing aid centers, cochlear implant centers, rehabilitation centers, and vestibular centers. Participation was entirely voluntary, and no incentives were provided to encourage participation. Before beginning the survey, all potential participants were presented with an ‘Informed Consent’ form, detailing the purpose of the study and ensuring that their participation would be completely anonymous. Data collection was performed via an online platform (Google Forms).

### 2.3. Data Collection

The Socio-Demographic Form, an eight-question form containing questions on socio-demographic information, was also completed by the participants. This form included questions about age, gender, education level, workplace, position, work experience, the number of audiologists in the workplace, and the number of patients per day.

The Maslach Burnout Inventory—General Form (MBI-GF) is a 5-point Likert scale consisting of 16 items and three subscales. The subscales consist of 5 items each for exhaustion, 5 for depersonalization, and 6 for competence. Scale items are scored on a scale from “never” to “always.” High scores on the exhaustion and depersonalization subscales and low scores on the competence (inversely scored) subscale indicate burnout. Three separate burnout scores were calculated for each individual. The test was adapted into Turkish and validated [9].

The Job Satisfaction Scale (JSS) consists of 3 sections and 25 questions. Each section contains 8 questions. The sections on career, management, and conditions comprise 24 questions designed to measure job satisfaction. The final question is open-ended. A 5-point Likert scale is used in the questionnaire. A 1–5 scale is used, where “Not satisfied at all” is represented by 1 and “Very satisfied “ by 5. The scale was prepared and validated [10].

### 2.4. Data Analysis and Interpretation

Statistical analyses were performed using SPSS 27.0 (IBM Corp., Armonk, NY, USA), and the Structural Equation Modeling (SEM) was conducted using AMOS 27.0 (IBM Corp., Wexford, PA, USA) software. Descriptive statistics, including frequencies and means, were calculated. Reliability was verified using Cronbach’s Alpha coefficients, which were found to be 0.847 for the MBI and 0.953 for JS. The distribution of data was assessed using the Kolmogorov–Smirnov test and skewness–kurtosis values. All values ranged between −1.5 and +1.5, indicating a normal distribution. Parametric tests (independent-sample *t*-test and ANOVA, with Tukey post hoc analysis) were used for group comparisons. Our final sample of 254 audiologists significantly exceeds the requirement, ensuring high statistical power and reducing the likelihood of Type II errors. Additionally, this sample size satisfies the rule of thumb for SEM, which suggests a minimum ratio of observations to variables of 10:1. The predictive relationships between variables were tested via SEM. Structural Equation Modeling (SEM) was preferred over traditional regression analysis because SEM allows for the simultaneous estimation of multiple and interrelated dependence relationships. Unlike regression, SEM accounts for measurement error in the observed variables, providing more accurate path coefficients. In our hypothesized model, the three sub-dimensions of the Maslach Burnout Scale (exhaustion, depersonalization, and competence) were defined as exogenous variables (predictors), while job satisfaction was defined as the endogenous variable (outcome). The model utilized Maximum Likelihood estimation. The SEM model was evaluated using a combination of absolute and incremental fit indices. The results indicated that the model provided a good fit to the observed data. The ratio of chi-square to degrees of freedom was found to be within the recommended range (x^2^/df = 2.14, *p* < 0.001). Other fit indices also supported the adequacy of the model, with a GFI of 0.93, an AGFI of 0.90, and a CFI of 0.96. Additionally, the RMSEA was calculated as 0.058 (90% CI: 0.045–0.072), which is below the threshold of 0.08, and the SRMR was 0.042. Collectively, these indices suggest that the proposed structural model effectively represents the relationships between burnout dimensions and job satisfaction among audiologists.

## 3. Results

### 3.1. Job Satisfaction Results

The analysis of job satisfaction revealed that demographic characteristics and professional roles are significant determinants of vocational fulfillment. Age, education level, professional experience, workplace setting, and job position emerged as the primary factors influencing overall satisfaction scores (Table 2).

Higher job satisfaction was consistently associated with advanced career stages and higher academic attainment. Audiologists aged 40 and older, those with post-graduate degrees (master’s or doctorate), and those with 6–10 years of experience reported significantly higher total satisfaction compared to younger (20–30), bachelor’s degree-holding, and early-career (1–5 years) colleagues. Specifically, significant differences were observed across all sub-dimensions (career, management, and conditions) for age and education groups (F_total_ = 7.391, *p* = 0.001, η_p_^2^ = 0.056). Gender differences were less pervasive, being limited to the management dimension, where male audiologists reported higher scores than females (d = 0.34, *p* = 0.007); no significant gender impact was found for career or conditions (*p* > 0.05).

The clinical setting and professional authority played a decisive role in satisfaction levels. Audiologists in employer positions demonstrated substantially higher satisfaction across all sub-dimensions compared to employees, representing a very large effect size (t = −4.737, *p* < 0.001, d = 1.15). Workplace type also yielded a significant impact (F = 7.531, *p* < 0.001, η_p_^2^ = 0.109); professionals in cochlear implant and hearing aid centers reported higher satisfaction with management and working conditions than those in rehabilitation centers. Regarding work environment density, facilities with two or more audiologists fostered higher satisfaction with management. Notably, managing a daily caseload of 10 or more patients was associated with higher scores in the conditions dimension (Table 2).

### 3.2. Burnout Syndrome Results

Individual and organizational factors were found to significantly influence burnout levels among audiologists. Age, professional experience, job position, and workplace staff density emerged as the most substantial predictors of burnout dimensions (Table 3).

The analysis revealed that age and professional seniority are inversely related to burnout. Audiologists in the 41+ age group and those with more than 6 years of experience reported significantly lower exhaustion and higher professional competence compared to their younger and less experienced counterparts (F_age_ = 15.493, *p* < 0.001, η_p_^2^ = 0.110; F_experience_ = 13.253, *p* < 0.001, η_p_^2^ = 0.095). In terms of gender, while female audiologists exhibited slightly higher exhaustion scores (d = 0.35, *p* = 0.007), gender did not significantly impact depersonalization or total burnout scores. Education level primarily influenced depersonalization, with master’s degree holders reporting lower scores than those with bachelor’s degrees.

The organizational setting played a critical role in professional well-being. Audiologists working in specialized centers (cochlear implant, hearing aid, and vestibular) reported more favorable scores in career and management dimensions compared to those in rehabilitation centers. Job position was another major factor; employers demonstrated significantly lower burnout levels than employees (d = 0.57, *p* = 0.021). Furthermore, the presence of colleagues served as a protective factor, as facilities with two or more audiologists recorded significantly lower exhaustion and depersonalization scores than solo-practitioner settings. Notably, the volume of daily patient care did not show a statistically significant relationship with any burnout dimensions (*p* > 0.05).

### 3.3. Correlation Analysis Results

A statistically significant and negative correlation was found between the scores obtained by audiologists from the total Maslach Burnout Scale (and its sub-dimensions: exhaustion and depersonalization) and the total Job Satisfaction Scale (*p* < 0.05). Based on Cohen’s criteria, these relationships represented large effect sizes. Depersonalization emerged as the factor with the most substantial impact on job satisfaction, showing a large negative effect size (r = −0.636), which was slightly higher than that of the exhaustion dimension (r = −0.625). The career sub-dimension also demonstrated very high negative correlations with both exhaustion (r = −0.608) and depersonalization (r = −0.606), further indicating a large effect magnitude. Conversely, a statistically significant and positive correlation was identified between the professional competence subscale of the Maslach Burnout Scale and total job satisfaction scores (*p* < 0.05). However, this relationship exhibited a small effect size (r = 0.162), suggesting that while the association is statistically significant, its practical impact on overall satisfaction is less pronounced than that of the negative burnout dimensions (Table 4).

### 3.4. Structural Equation Modeling (SEM) Results

**Figure 1:**
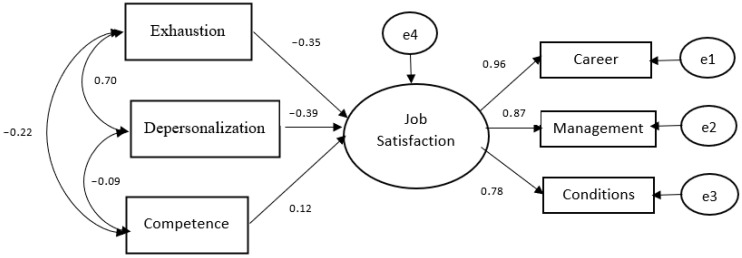
Prediction of Maslach Burnout Scale scores of audiologists based on Job Satisfaction Scale scores. Note: e1–e4 represent error terms (residual variances).

The Structural Equation Modeling (SEM) analysis revealed that all three dimensions of the Maslach Burnout Scale were significant predictors of job satisfaction, collectively accounting for a substantial 52% of the variance in Job Satisfaction scores (R^2^ = 0.52). This represents a large effect size for the overall model. Specifically, depersonalization emerged as the strongest negative predictor of job satisfaction (β = −0.39; *p* < 0.001), representing a medium to large effect. Similarly, exhaustion significantly and negatively predicted job satisfaction (β = −0.35; *p* < 0.001), also demonstrating a medium effect size. In contrast, while the competence subscale significantly and positively predicted job satisfaction scores (β = 0.12; *p* = 0.010), this relationship exhibited a small effect size. These findings indicate that while all dimensions are statistically significant, the negative impacts of depersonalization and exhaustion on professional well-being are substantially more pronounced than the positive contribution of professional competence. Furthermore, the model explained 38% of the variance in overall burnout symptoms (R^2^ = 0.38), highlighting the strong predictive power of the organizational and individual factors integrated into the structural framework (Figure 1).

## 4. Discussion

The primary objective of this study was to provide a structural understanding of the factors influencing the professional well-being of audiologists in Türkiye. By employing Structural Equation Modeling (SEM), this research investigates the predictive relationships between burnout sub-dimensions and job satisfaction components, moving beyond descriptive prevalence. Our findings suggest that professional dissatisfaction among audiologists is statistically associated with perceived organizational challenges rather than individual characteristics. This framework underscores that professional well-being appears to be linked to structural factors such as workload and the occupational environment. Consequently, this study shifts the discourse toward the importance of organizational improvements, offering evidence-based insights into the systemic nature of burnout and job satisfaction within the profession.

The socio-demographic and occupational characteristics of audiologists appear to play a role in shaping their levels of burnout and job satisfaction. In terms of age and professional experience, our observations suggest a tendency for older audiologists to report lower levels of burnout compared to their younger colleagues. It could be argued that younger employees may initially experience dissatisfaction due to a perceived gap between academic expectations and clinical realities [11]; however, this gap seems to narrow as professional experience potentially fosters more realistic perspectives. As noted by Brännström et al. [12], increasing age and experience may act as protective factors, as seasoned audiologists might develop higher efficiency in clinical tasks, which is often thought to be associated with lower stress perception. Furthermore, long-term employment is often associated with factors such as seniority, which could potentially support higher satisfaction [13]. Nevertheless, the literature remains divided, with some studies reporting no significant differences [8] or even an increased risk of burnout with age [14,15]. This divergence suggests that any protective effect may be linked not merely to chronological age, but perhaps more closely to the clinical mastery and perceived competence acquired over time.

Gender-based differences also emerge as an important area of analysis. In the current study, male audiologists tended to report higher job satisfaction, while female audiologists demonstrated a tendency toward higher burnout scores. Although some of the literature finds no significant difference [8], our results appear to align with studies that have identified significant gender-based disparities [16]. These variations could potentially be associated with broader social and emotional factors, as well as differing workplace perceptions [17]. However, these findings should be interpreted with caution, as they may reflect the specific socio-demographic context of this cohort rather than a universal professional trend.

Education level appears to be a significant factor associated with professional well-being. Our findings indicate that postgraduate education is linked to higher levels of job satisfaction and lower burnout scores. While Mobaraki [8] did not observe a significant effect of education and Celikgün et al. [18] noted that undergraduate graduates reported higher satisfaction, our data suggest a different trend. Advanced education may benefit professionals not just through the degree, but by reinforcing their self-confidence and internal sense of competence. However, as these psychological mediators were not directly measured, this relationship remains a subject for further qualitative exploration.

The clinical environment appears to be a significant factor in professional well-being, with rehabilitation centers presenting a complex profile of challenges. In this study, only 42.5% of audiologists reported satisfaction, which may be attributable to the emotional burden of pediatric care and frustrations associated with gradual clinical progress [14]. While some of the literature suggests higher emotional stability in these settings [18], other research documents lower job satisfaction in rehabilitation centers and private hospitals [19]. The complexities of this environment are further underscored by perceived role conflicts [20] and evidence indicating that a substantial portion of audiologists in these centers may belong to high-risk groups for occupational stress [21]. Conversely, cochlear implant centers reported higher satisfaction levels. This may stem from the multidisciplinary work environment and the motivation of witnessing tangible improvements in patients’ quality of life. In contrast, the lower competency scores observed in hearing aid centers suggest a risk of professional stagnation. These variations across settings highlight that professional well-being is closely linked to the specific organizational context [22], as reflected in the participants’ reported experiences, even where institutional structures were not directly audited.

Structural and organizational factors, including job position, team size, and patient flow, appear to be essential components of professional well-being [23]. Employer audiologists tended to report higher satisfaction and lower burnout levels, which could potentially be associated with the greater autonomy and control they perceive over their work environment. These factors often appear to be less accessible for employee audiologists, who frequently operate within more rigid protocols [24].

Regarding social support, workplaces with two or more audiologists may facilitate workload sharing, a factor associated with reduced isolation and stress in this cohort [25,26]. Consistent with Saldırım et al. [27], an increase in the number of audiologists within a facility tended to correlate with lower stress scores. Similarly, higher patient volume (10+ patients/day) was associated with better ‘conditions’ scores; this could potentially be attributed to the possibility that high-volume facilities provide more robust organizational structures and support staff [28]. These findings suggest that professional well-being is closely related to perceived organizational support and structural resources [29,30], although these systemic factors were not directly measured as independent constructs in the current study.

### General Assessment of Job Satisfaction and Burnout

In this study, 59.73% of audiologists reported job satisfaction, while 32.43% exhibited signs of burnout. These findings suggest that approximately one in three audiologists in Türkiye may be experiencing burnout, with a significant portion of the workforce reporting only moderate or partial satisfaction. Our results appear to be congruent with existing research in the Turkish context; for instance, Celikgün et al. [18] documented similarly low satisfaction levels, while Köroğlu et al. [21] and Saldırım et al. [27] characterized the profession as a high-risk group for occupational stress. Although Cengiz et al. [19] reported high self-esteem scores, suggesting that audiologists inherently value their profession, our data suggest that systemic workplace challenges may negatively impact the expression of this vocational passion.

When compared with the international literature, the professional well-being profile of audiologists in Türkiye presents distinct variations. While studies in countries such as New Zealand [15], India [14], Iran [8], and the United States [7] have reported relatively high satisfaction and low burnout levels, the findings in this Turkish cohort appear to align more closely with the occupational stress indicators observed in the United Kingdom [6] and Portugal [31]. These results suggest that burnout and fulfillment are driven by specific organizational contexts and workloads, rather than being inherent to the audiology profession. Consequently, these findings highlight the importance of addressing workplace-specific stressors to support the long-term well-being of the profession [7].

Our analysis reveals a robust inverse relationship between occupational burnout and job satisfaction, indicating that these constructs function as opposing markers of professional well-being among audiologists. Statistically significant negative correlations demonstrate that as feelings of exhaustion and depersonalization increase, job satisfaction tends to diminish. Notably, depersonalization emerged as a critical factor, exhibiting a stronger negative association with job satisfaction (r = −0.636) than emotional exhaustion (r = −0.625). This suggests that psychological detachment from patients and clinical practice appears to be a primary correlate of reduced professional enjoyment [29]. Furthermore, the strong negative correlation with working conditions (r = −0.624) indicates that the work environment is closely associated with professional alienation. This erosion of well-being extends to career perspectives, as evidenced by the high negative correlations between the career subscale and markers of burnout (exhaustion: r = −0.608; depersonalization: r = −0.606). These findings highlight that chronic burnout is proportionally linked to diminished long-term career aspirations. Conversely, the competence subscale presents a distinct dynamic. While professional competence maintains a positive, albeit modest, correlation with job satisfaction (r = 0.162), its lack of correlation with working conditions (*p* = 0.562) suggests that an audiologist’s confidence in their clinical skills may remain independent of their physical work environment. While this internal sense of efficacy is valuable, our findings suggest it may be insufficient on its own to buffer against the broader impact of burnout. Finally, the role of professional maturity was evident, as increasing age and experience were consistently linked to higher satisfaction and lower burnout, reinforcing the potential benefit of targeted interventions to support less experienced audiologists [30].

Our structural equation model identified that the impersonal approach emerged as a significant negative predictor of job satisfaction (β = −0.39). This suggests that when audiologists perceive their patients primarily as cases within a heavy workload, their professional fulfillment may tend to diminish. This trend could potentially undermine career perception (0.96), which was identified as the strongest indicator of job satisfaction in this study. This might imply that professional advancement is sustained not only by technical proficiency but also by the quality of the empathetic connection established with patients [8]. Furthermore, the occupational environment characterized by factors such as long hours in soundproof booths is statistically associated with increased burnout scores (β = −0.35). These burnout levels appear to be linked to job satisfaction through organizational conditions (0.78), potentially making the physical challenges of the work environment perceived as more burdensome [7]. Management (0.87), another robust component of job satisfaction, may function as a protective buffer; our findings suggest that when audiologists perceive support from their administration, they might maintain higher professional commitment despite burnout symptoms. Finally, while professional competence is positively associated with satisfaction, this effect was relatively weak (β = 0.12). This underscores the possibility that clinical mastery alone may not be sufficient to ensure well-being when faced with adverse organizational management or significant emotional exhaustion [13].

While this study provides significant insights into the professional well-being of audiologists in Türkiye, several limitations should be considered when interpreting the results. First, the cross-sectional design of the study provides a snapshot of participants’ experiences at a single point in time, precluding the establishment of causal relationships or the observation of long-term trends. Future research employing longitudinal designs will be necessary to capture the dynamic nature of burnout over the course of a professional career. Second, online convenience and snowball sampling may limit generalizability. A potential non-response bias exists, as highly burnt-out individuals might have been less likely to participate. Third, the relatively small sample size in certain subgroups, such as employer audiologists, may affect the statistical stability of some estimates and should be interpreted with caution. Finally, the reliance on self-reported measures for burnout and job satisfaction, while standard in this field, is subject to social desirability and subjective perception. Despite these limitations, this study offers a robust foundation for understanding the systemic factors influencing the audiology profession and provides a clear direction for necessary institutional reforms.

Moving forward, the path to a resilient professional future lies in shifting the focus from individual resilience to corporate responsibility through supportive governance and the protection of professional autonomy. Methodologically, this study underscores a critical transition in health workforce research: moving beyond descriptive burnout prevalence toward structural predictive frameworks. By employing Structural Equation Modeling (SEM), we have provided a more nuanced understanding of how systemic variables interact to shape professional well-being, positioning this research within the evolving trend of evidence-based health management. Future research could build upon this SEM-based framework by prioritizing longitudinal studies to track the evolution of audiologists’ career stages over time. Future qualitative or mixed-methods research could better explore nuanced challenges in high-risk settings, such as rehabilitation centers, where quantitative metrics may miss structural stressors. Notably, as healthcare continues to digitize, future studies should also integrate emerging methodologies such as Natural Language Processing (NLP) and the semantic mapping of professional activities. Analyzing clinical documentation through these advanced techniques could provide more objective insights into the interplay between administrative workload and emotional exhaustion [32]. Finally, investigating the long-term impacts of these evolving healthcare models through advanced structural modeling will be critical to ensuring a sustainable and fulfilling career for audiologists.

## 5. Conclusions

The findings of this study highlight that burnout and job satisfaction among audiologists are not merely reflections of technical proficiency but are significantly linked to the interplay between emotional labor and organizational dynamics. As a profession requiring constant human interaction, audiology involves a substantial emotional component that appears to influence professional well-being. Our results suggest that burnout symptoms are closely associated with perceived systemic challenges, such as organizational conditions and a lack of administrative support, rather than being a reflection of personal inadequacy. While individual growth is important, our findings indicate that enhancing professional well-being primarily requires improving the organizational environment. Consequently, addressing these structural factors emerges as a critical requirement for supporting the long-term sustainability of the audiology workforce.

## Figures and Tables

**Table 1:** Distribution of audiologists’ socio-demographic characteristics.

	Number (*n*)	Percentage (%)
Age		
20–30	133	52.40
31–40	82	32.30
41+	39	15.40
Gender		
Female	152	59.80
Male	102	40.20
Education level		
Bachelor	162	63.80
Master	59	23.20
Doctorate	33	13.00
Workplace		
Hospital	39	15.40
Hearing aid center	93	36.60
Cochlear implant center	32	12.60
Rehabilitation center	60	23.60
Vestibular center	30	11.80
Position		
Employee	236	92.90
Employer	18	7.10
Work Experience		
1–5 years	120	47.20
6–10 years	90	35.40
11+ years	44	17.30
Number of audiologists in place		
1	49	19:30
2	108	42.50
3+	97	38.20
Number of patients per day		
1–3 patients	38	15.00
4–6 patients	101	39.80
7–9 patients	64	25.20
10+ patients	51	20.10
Total	254	100.00

**Table 2:** Job satisfaction results for audiologists.

		Career		*p*	Management		*p*	Conditions		*p*	Total		*p*
		Mean ± SD	t/F		Mean ± SD	t/F		Mean ± SD	t/F		Mean ± SD	t/F	
Age	20–30 (*n* = 133)	3.57 ± 0.57	8.753 (F)	0.000 *** (1–3)	3.46 ± 0.66	8.021 (F)	0.000 * (1–3)	3.28 ± 0.55	3.680 (F)	0.027 * (1–3)	3.44 ± 0.54	5.742 (F)	0.004 ** (1–3)
	31–40 (*n* = 82)	3.72 ± 0.49			3.67 ± 0.61			3.15 ± 0.53			3.51 ± 0.51		
	41+ (*n* = 39)	3.95 ± 0.31			3.88 ± 0.34			3.41 ± 0.35			3.75 ± 0.29		
Gender	Female (*n* = 133)	3.62 ± 0.52	−1.932 (t)	0.054	3.51 ± 0.63	−2.742 (t)	0.007 **	3.22 ± 0.53	−1.430 (t)	0.154	3.45 ± 0.50	−2.276 (t)	0.024 *
	Male (*n* = 133)	3.75 ± 0.54			3.72 ± 0.59			3.32 ± 0.50			3.60 ± 0.51		
Education Level	Bachelor (*n* = 162)	3.57 ± 0.56	9.637 (F)	0.000 *** (1–2)	3.51 ± 0.66	4.103 (F)	0.018 * (1–2)	3.18 ± 0.54	6.546 (F)	0.002 ** (1–2)	3.42 ± 0.53	7.391 (F)	0.001 *** (1–2)
	Master (*n* = 59)	3.82 ± 0.35		(1–3)	3.73 ± 0.43		(1–3)	3.45 ± 0.34		(1–3)	3.67 ± 0.32		(1–3)
	Doctorate (*n* = 33)	3.93 ± 0.54			3.75 ± 0.68			3.34 ± 0.62			3.67 ± 0.57		
Workplace	Hospital (*n* = 39)	3.73 ± 0.51	6.920 (F)	0.000 *** (3–4)	3.43 ± 0.71	4.959 (F)	0.001 *** (2–4)	3.26 ± 0.54	9.227 (F)	0.000 *** (2–4)	3.47 ± 0.54	7.531 (F)	0.000 *** (2–4)
	Hearing Aid Center (*n* = 93)	3.74 ± 0.55		(4–5)	3.71 ± 0.61		(3–4)	3.41 ± 0.46		(3–4)	3.62 ± 0.50		(3–4)
	Cochlear Implant Center (*n* = 32)	3.85 ± 0.34			3.75 ± 0.50		(4–5)	3.40 ± 0.40			3.67 ± 0.34		
	Rehabilitation Center (*n* = 60)	3.38 ± 0.50			3.36 ± 0.56			2.94 ± 0.52			3.23 ± 0.47		
	Vestibular Center (*n* = 30)	3.81 ± 0.55			3.75 ± 0.59			3.28 ± 0.53			3.61 ± 0.52		
Position	Employee (*n* = 236)	3.64 ± 0.52	−4.419 (t)	0.000 ***	3.54 ± 0.60	−4.691 (t)	0.000 ***	3.23 ± 0.51	−3.693 (t)	0.000 ***	3.47 ± 0.49	−4.737 (t)	0.000 ***
	Employer (*n* = 18)	4.19 ± 0.44			4.23 ± 0.49			3.69 ± 0.53			4.04 ± 0.44		
Work Experience	1–5 Years (*n* = 120)	3.53 ± 0.56	9.556 (F)	0.000 *** (1–2)	3.42 ± 0.63	10.173 (F)	0.000 *** (1–2)	3.26 ± 0.55	0.231 (F)	0.794	3.40 ± 0.52	5.647 (F)	0.004 ** (1–2)
	6–10 Years (*n* = 90)	3.81 ± 0.50		(1–3)	3.78 ± 0.56		(1–3)	3.29 ± 0.52			3.63 ± 0.50		
	11+ Years (*n* = 44)	3.81 ± 0.40			3.68 ± 0.58			3.22 ± 0.45			3.57 ± 0.44		
Number of audiologists in place	1 (*n* = 49)	3.63 ± 0.67	0.187 (F)	0.830	3.38 ± 0.81	3.845 (F)	0.023 * (1–2)	3.24 ± 0.61	0.048 (F)	0.953	3.42 ± 0.64	0.999 (F)	0.370
	2 (*n* = 108)	3.69 ± 0.52			3.64 ± 0.58		(1–3)	3.27 ± 0.49			3.53–0.48		
	3+ (*n* = 97)	3.69 ± 0.47			3.65 ± 0.53			3.26 ± 0.51			3.53–0.47		
Number of patients per day	1–3 Patients (*n* = 38)	3.66 ± 0.53	2.531 (F)	0.058	3.70 ± 0.56	1.233 (F)	0.298	3.17 ± 0.47	2.707 (F)	0.046 * (1–4)	3.51 ± 0.48	1.673 (F)	0.173
	4–6 Patients (*n* = 101)	3.62 ± 0.50			3.58 ± 0.58			3.20 ± 0.48			3.46 ± 0.47		
	7–9 Patients (*n* = 64)	3.64 ± 0.54			3.49 ± 0.67			3.29 ± 0.57			3.48 ± 0.53		
	10+ Patients (*n* = 51)	3.86 ± 0.56			3.67 ± 0.67			3.42 ± 0.55			3.65 ± 0.55		

Note: Comparative analysis was performed using an independent-sample *t*-test (t) for gender and position; one-way ANOVA (F) was performed for age, education level, workplace, work experience, number of audiologists and patients per day. * *p* < 0.05, ** *p* < 0.01, *** *p* < 0.001.

**Table 3:** Burnout syndrome results for audiologists.

		Exhaustion		*p*	Depersonalization		*p*	Competence		*p*	Total		*p*
		Mean ± SD	t/F		Mean ± SD	t/F		Mean ± SD	t/F		Mean ± SD	t/F	
Age	20–30 (*n* = 133)	11.38 ± 3.21	9.643 (F)	0.000 *** (1–3)	10.21 ± 2.99	3.182 (F)	0.043 * (1–3)	20.83 ± 4.57	14.224 (F)	0.000 *** (1–3)	36.82 ± 7.79	15.493 (F)	0.000 *** (1–3)
	31–40 (*n* = 82)	10.39 ± 2.96			10.06 ± 2.68			23.40 ± 3.25			33.05 ± 6.39		
	41+ (*n* = 39)	9.10 ± 1.73			8.95 ± 2.13			23.64 ± 2.98			30.41 ± 5.43		
Gender	Female (*n* = 133)	11.13 ± 3.13	2.730 (t)	0.007 **	10.22 ± 2.98	1.781 (t)	0.076	22.19 ± 4.37	0.467 (t)	0.641	35.22 ± 7.66	1.572 (t)	0.117
	Male (*n* = 133)	10.08 ± 2.83			9.59 ± 2.47			21.94 ± 3.86			33.73 ± 7.04		
Education Level	Bachelor (*n* = 162)	10.92 ± 3.18	1.074 (F)	0.343	10.31 ± 2.84	4.485 (F)	0.012 * (1–2)	21.64 ± 4.27	2.780 (F)	0.064	35.64 ± 7.55	4.399 (F)	0.013 ** (1–2)
	Master (*n* = 59)	10.36 ± 2.70			9.05 ± 2.27			22.76 ± 3.73			32.64 ± 6.67		
	Doctorate (*n* = 33)	10.30 ± 3.01			9.94 ± 3.14			23.12 ± 4.17			33.12 ± 7.38		
Workplace	Hospital (*n* = 39)	11.41 ± 2.97	3.229 (F)	0.013 * (1–3)	9.95 ± 2.73	7.862 (F)	0.000 *** (1–3)	23.08 ± 3.83	3.527 (F)	0.008 ** (1–2)	34.31 ± 7.11	2.103 (F)	0.081
	Hearing Aid Center (*n* = 93)	10.42 ± 3.08		(3–4)	9.41 ± 2.62		(3–4)	20.85 ± 4.64		(2–3)	35.04 ± 8.46		
	Cochlear Implant Center (*n* = 32)	9.72 ± 2.48			8.97 ± 1.71			22.69 ± 3.86		(2–4)	32.00 ± 4.68		
	Rehabilitation Center (*n* = 60)	11.57 ± 3.06			11.58 ± 0.88			22.93 ± 3.33		(2–5)	36.25 ± 6.56		
	Vestibular Center (*n* = 30)	10.03 ± 3.15			9.57 ± 3.02			22.37 ± 4.16			33.23 ± 7.90		
Position	Employee (*n* = 236)	10.87 ± 3.06	3.160 (t)	0.002 **	10.10 ± 2.82	2.781 (t)	0.006 **	22.09 ± 4.06	0.037 (t)	0.971	34.92 ± 7.37	2.326 (t)	0.021 *
	Employer (*n* = 18)	8.56 ± 1.98			8.22 ± 1.86			22.06 ± 5.53			30.72 ± 7.39		
Work Experience	1–5 Years (*n* = 120)	11.57 ± 3.15	9.601 (F)	0.000 *** (1–2)	10.14 ± 3.00	0.434 (F)	0.648	20.73 ± 4.53	13.233 (F)	0.000 *** (1–2)	37.04 ± 8.00	13.253 (F)	0.000 *** (1–2)
	6–10 Years (*n* = 90)	9.93 ± 2.85		(1–3)	9.82 ± 2.63			23.30 ± 3.57		(1–3)	32.46 ± 6.20		(1–3)
	11+ Years (*n* = 44)	9.95 ± 2.57			9.80 ± 2.60			23.32 ± 3.03			32.43 ± 6.13		
Number of audiologists in place	1 (*n* = 49)	12.37 ± 3.41	9.622 (F)	0.000 *** (1–2)	10.94 ± 3.52	3.817 (F)	0.023 * (1–2)	21.20 ± 5.07	2.427 (F)	0.090	38.22 ± 9.11	8.137 (F)	0.000 *** (1–2)
	2 (*n* = 108)	10.37 ± 2.91		(1–3)	9.81 ± 2.53		(1–3)	21.91 ± 3.99			34.28 ± 7.10		(1–3)
	3+ (*n* = 97)	10.25 ± 2.75			9.65 ± 2.59			22.74 ± 3.78			33.18 ± 6.26		
Number of patients per day	1–3 Patients (*n* = 38)	10.21 ± 3.26	0.957 (F)	0.414	10.24 ± 2.90	0.898 (F)	0.443	22.79 ± 2.80	1.629 (F)	0.183	33.82 ± 7.54	0.924 (F)	0.430
	4–6 Patients (*n* = 101)	10.51 ± 2.87			10.16 ± 2.61			21.93 ± 4.00			34.74 ± 7.25		
	7–9 Patients (*n* = 64)	11.11 ± 3.00			9.94 ± 2.99			21.34 ± 4.41			35.70 ± 7.42		
	10+ Patients (*n* = 51)	10.96 ± 3.30			9.43 ± 2.86			22.82 ± 4.89			33.61 ± 7.74		

Note: Comparative analysis was performed using an independent-sample *t*-test (t) for gender and position; one-way ANOVA (F) was performed for age, education level, workplace, work experience, number of audiologists and patients per day. * *p* < 0.05, ** *p* < 0.01, *** *p* < 0.001.

**Table 4:** Correlations between Maslach Burnout Scale scores and Job Satisfaction Scale scores of Audiologists.

		Career	Management	Conditions	Job Satisfaction Scale
Exhaustion	r	−0.608 **	−0.596 **	−0.500 **	−0.625 **
	*p*	0.000	0.000	0.000	0.000
	*n*	254	254	254	254
Depersonalization	r	−0.606 **	−0.522 **	−0.624 **	−0.636 **
	*p*	0.000	0.000	0.000	0.000
	*n*	254	254	254	254
Competence	r	258 **	208 **	−0.037	0.162 **
	*p*	0.000	0.001	0.562	0.010
	*n*	254	254	254	254
Maslach Burnout Scale	r	−0.628 **	−0.562 **	−0.426 **	−0.592 **
	*p*	0.000	0.000	0.000	0.000
	*n*	254	254	254	254

Note: ** Correlation is significant at the 0.01 level (2-tailed).

## Data Availability

The data presented in this study are available on request from the corresponding author due to privacy and ethical restrictions regarding participant confidentiality.

## Abbreviations

The following abbreviations are used in this manuscript:

BS	Burnout syndrome
MBI-GF	Maslach Burnout Inventory—General Form
JS	Job satisfaction
JSS	Job Satisfaction Scale
SEM	Structural Equation Modeling
CFI	Comparative Fit Index
TLI	Tucker–Lewis Index
RMSEA	Root Mean Square Error of Approximation
SRMR	Standardized Root Mean Square Residual
GFI	Goodness of Fit Index
NLP	Natural Language Processing
